# A novel aggregated coefficient ranking based feature selection strategy for enhancing the diagnosis of breast cancer classification using machine learning

**DOI:** 10.1038/s41598-025-87826-7

**Published:** 2025-02-04

**Authors:** E. Sreehari, L. D. Dhinesh Babu

**Affiliations:** School of Computer Science Engineering and Information Systems, Vellore Institute of Technology, Vellore, Tamil Nadu 632014 India

**Keywords:** Computational biology and bioinformatics, Health care, Oncology, Mathematics and computing

## Abstract

Effective Breast cancer (BC) analysis is crucial for early prognosis, controlling cancer recurrence, timely medical intervention, and determining appropriate treatment procedures. Additionally, it plays a significant role in optimizing mortality rates among women with breast cancer and increasing the average lifespan of patients. This can be achieved by performing effective critical feature analysis of the BC by picking superlative features through significant ranking-based Feature Selection (FS). Various authors have developed strategies relying on single FS, but this approach may not yield excellent results and could lead to various consequences, including time and storage complexity issues, inaccurate results, poor decision-making, and difficult interpretation of models. Therefore, critical data analysis can facilitate the development of a robust ranking methodology for effective feature selection. To solve these problems, this paper suggests a new method called Aggregated Coefficient Ranking-based Feature Selection (ACRFS), which is based on tri chracteristic behavioral criteria. This strategy aims to significantly improve the ranking for an effective Attribute Subset Selection (ASSS). The proposed method utilized computational problem solvers such as chi-square, mutual information, correlation, and rank-dense methods. The work implemented the introduced methodology using Wisconsin-based breast cancer data and applied the Synthetic Minority Oversampling Technique (SMOTE) to the obtained data subset. Later, we employed models such as decision trees, support vector machines, k-nearest neighbors, random forests, stochastic gradient descent, and Gaussian naive bayes to determine the type of cancer. The classification metrics such as accuracy, precision, recall, F1 score, kappa score, and Matthews coefficient were utilized to evaluate the effectiveness of the suggested ACRFS approach. The proposed method has demonstrated superior outcomes with fewer features and a minimal time complexity.

## Introduction

Breast cancer (BC) is a global health concern that affects millions of women worldwide. It is the leading cause of death worldwide, resulting in millions of deaths every year, accounting for almost 1 in 6 deaths. Globally, the most prevalent cancers are breast cancer (15%), lung cancer (13%), bowel cancer (11%), and prostate cancer (14%). According to CANCER-RESEARCH-UK, the number of deaths from these four cancers surpasses the total number of cancers diagnosed worldwide^1^. GLOBOCON has identified 36 cancer types in 185 countries worldwide, out of the over 100 documented cancer types. The latest reports from the WHO indicate a rise in cancer-based burden deaths to 19.3 million new cases, up from 10 million deaths in 2020^2^. From 1990 to 2019, there was a potential increase of 75%, but from 2020 to 2024 alone, it has invariably doubled^3,4^.

**Fig. 1:**
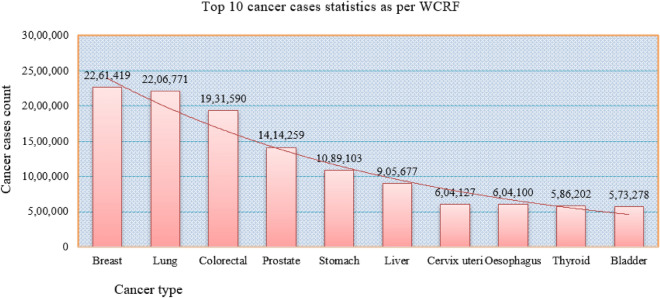
The impact of top 10 cancer type statistics globally.

As shown in Fig. 1, the statistics indicate that BC is the leading cause of cancer deaths globally. Understanding the treatment timeline for inflammatory breast cancer, from diagnosis to recovery, requires careful analysis and diagnosis. Early detection and timely medical intervention can improve the prognosis and greatly increase the survival rate, as well as playing a major role in managing and treating cancer. Furthermore, the precise categorization of benign tumors might avert patients from undertaking unneeded medical interventions. Therefore, researchers are dedicating extensive resources to accurately diagnose breast cancer and classify cancer patients into either the malignant or benign category. Combining Machine Learning (ML) approaches with crucial feature analysis is an effective and useful approach for data classification, particularly when applied to cancer datasets. Strong informative features in the data can have a significant, unbiased impact. Furthermore, the rationale for employing critical feature analysis stemmed from the realization that incorporating all features could result in performance, escalate the model’s temporal and spatial complexity, and potentially complicate its interpretation. Support for feature selection is critical and plays a significant and vital role in multidisciplinary fields. Most of the authors did rankings for features and explored using the single FS method based on coefficient determined. This kind of strategy would lead to many consequences. Despite the significant emphasis on ranking-based FS for BC analysis using single FS ranking techniques in previous works, there has been little priority given to FS-based combined ranking for breast cancer analysis.Using one method is not a good strategy to figure out the FS with computed coefficients and make a ranking schema. A strategy is required that performs well with both linear and nonlinear data. So, considering the FS-based ranking would assist us in identifying the best features, which are strongly and weekly relevant by their nature^5,6^. A ranking strategy for determining effective features would help us implement the model with less computational complexity, robust accuracy, and easy interpretation for overall analysis. To identify robust quality features, we proposed ACRFS technique, which employs three problem solvers such as mutual information, correlation coefficient, and Chi square to rank features. ACRFS, along with the Synthetic Minority Oversampling Technique (SMOTE) balancing technique^7,8^, and ML strategies, can help to identify superlative critical analysis by reducing the number of features in order to improve the diagnosis accuracy.

Effective execution of the proposed system requires a focus on the following objectives:

- To conduct a comprehensive examination and analysis of various studies carried out by different authors, relevant to the use of coefficient-based ranking methods in FS analysis for breast cancer analysis.
- To create a novel ACRFS strategy that will facilitate accurate determination and ranking of coefficients. Through this, we seek to identify the most influential features that contribute to robust diagnostic outcomes for breast cancer.
- To utilize SMOTE for balancing, look at the different FS-related ranking strategies and use various ML models, such as RF, DT, SGD, KNN, GNB, and SVM, on the constructed subset that was made using the ACRFS strategy.
- To assess the efficacy of the suggested approach by employing performance metrics like accuracy, precision, recall, F1 score, Kappa score, and MCC metrics.
- Examine the proposed ACRFS methodology capability determination levels, taking into account its time complexity and constraints.

The structure of the manuscript follows this format: In Section 2, we talk about related work. Section 3 delves into the overall implementation architectural procedure, outlines the proposed methodology workflow and its algorithm, and provides information on the data considered for analysis. Section 4 details the different ML models and their comparative analysis, comparing them before and after feature selection using distinct classification metrics. This section also covers the limitations and time complexity of the suggested concept. Section 5 will provide a conclusion and outline future work.

## Related work

Several risk factors, including an unhealthy lifestyle, genetic factors, psychosocial aspects, tobacco and alcohol consumption, a family history of BC, and specific medical disorders, primarily cause BC, according to the first preliminary study^9^. This section provided a thorough study and detailed analysis of different methodologies shown in Table 1 and it’s continued part relevant to BC analysis and feature selection. The works from 2019 to 2023 are considered to conduct a thorough investigation, focusing on BC as a comprehensive approach to critical analysis. We acknowledged shortcomings and sought to gain insight into the extent of information on BC by articulating relevant works during this specific period. We collected, examined, scrutinized, and deliberated upon the works using two main criteria: ranking-based FS and critical analysis of BC.

**Table 1:** The studied relevant works of literature concerning FS and BC analysis were studied.

Reference	Objective	methods used	Data set	Metrics	Results	Limitations
Ping Qiu and Niu.^10^	TCIC FS strategy for high dimensional data	SVM, KNN, DT, NB	ALL, colon, Gastric, Leuk, Lymp, Aden, Myel, Pros	Accuracy, sensitivity, specificity	Obtained low complexity, works well on All-Gastric-Leuk-Lymp	Suggested to work on user modeling for knowledge extraction
Reza Rabiei et al.^11^	Predicting BC using ML strategies	RF, MLP, GBT, GA for FS	Motamed cancer institute Tehran, Iran	AUC, Sensitivity, Specificity, Accuracy	RF obtained 80% accuracy, 95% sensitivity, 80% specificity	Modeling based on data from a single database and lacking genetic information
Shaode et al.^12^	Stable feature ranking algorithm for BC diagnosis	ANN, KNN, LDA, SVM	Four data sets BCDR, WBCD, GSE10810, GSE15852	AUC, Sensitivity, Specificity, Accuracy	Results identified three algorithms achieved good stability with $\ge {0.55}$	The proposed method restricted to the input of quantitative features.
Shaode et al.^13^	Stability compute evaluation of feature ranking algorithms	23 Feature ranking algorithms	Medical, gene imaging BCDR-F03, WBCD, GSE10810, GSE15852 sets	Advanced stability estimator	GFS, PWFP, and LNEC are identified as consistently exhibiting good stability	Explored the stability of ranking using one estimator, which may not be a good idea
Chun-jiang Tian et al.^14^	Evaluating FS methods using Mammographic data	correlation, LASSO, Laplacian, UFSOL, WILCOXON	Digital database for screening mammography	Accuracy, sensitivity, specificity	Proposed method obtained accuracy of 85%, 86%	They only applied a single classifier to small set
Eskandar Taghizadeh et al.^15^	BC prediction with FS and ML	ANOVA, MI, LR, Extra Tree classifier FS, 13 classification methods	TCGA database	Accuracy, sensitivity, specificity	FS LGR+MLP classifier achieved accuracy, AUC 0.86, 0.94	Authors not provided a comprehensive analysis of the limitations and future scope of the proposed method
Sara Ibrahim et al.^16^	BC diagnosis using correlation analysis and PCA	LR, SVM, KNN, DT, NB, RF, EC, hard and soft voting	WBCD dataset	Accuracy, Precision, Recall, F-Measure	Achieved an accuracy 98.24%, precision 99.29% recall 95.89%	Authors suggested to work with deep learning for classification
N C Lopez et al.^17^	To evaluate different FS techniques using BC data	Pearson, Relief, RFE with SVM	Breast cancer MCC Spain data	AUC	SVM-RFE ranking technique turned out to be highly stable	Directed to focus ensemble strategies to increase the FS methods stability
Mahendran Botlagunta et al.^18^	BC classification	LR, SVM, KNN, RF, DT, GB, XGB	Basavatarakam Indo-American Cancer Hospital & Research Institute BC data	Accuracy and AUC	DT classifier showed better accuracy (83%), AUC (87%), and scores	suggested to create a precise blood platform to improve survival and minimize medical costs.
Chour Singh Rajpoot et al.^19^	BC analysis using FS	GA, ant colony optimization, HHNN-E2SAT	WBCD dataset	Accuracy, F1-score, Sensitivity, Specificity, Precision	HHNN E2SAT achieved an accuracy of 98%	Authors used only one FS mechanism
Moloud Abdar et al.^20^	Ensemble model for enhancing the BC diagnosis	Bayes Net and NB methods	WBCD dataset	accuracy, precision, recall, F1score	SVM-NB-Metaclassifier shown more efficiency	Didn’t given much priority for FS
Ganjar Alfian et al.^21^	BC Risk Factors Using SVM and Extra-Trees	XGBoost, AdaBoost, MLP, SVM, LR, KNN, DT, NB, RF	Gynaecology University Hospital Coimbra from 2009-13	Accuracy, Precision, Sensitivity, Recall	The proposed model improved diagnostic decision systems	Small set of population and less priority of FS
Rasool A et al.^22^	BC Diagnosis using ML models	KNN, SVM, LR, and ensemble classifier	WDBC and BC Coimbra datasets	Accuracy, Precision, Recall, Fscore	The LR with RFE was able to achieve 98% accuracy and 97% F1-score	The authors didn’t focus on estimating computational complexity
Muhammet Fatih A^23^	BC diagnosis based on ML	SVM, KNN, RF, SVC, DT, LR	WBCD data	Accuracy,	LR achieved an approximate 98% accuracy	No priority given for FS approach

Ping Qiu and Zhendong Niu^10^ implemented a strategy called Total Correlation Information Coefficient (TCIC) based FS to avoid setting hyperparameters and optimal feature selection. The authors estimated associations among multiple variables using Gaussian copula and the total correlation-based information coefficient and then applied them to nine different kinds of data sets. To test how well their suggested method works, the authors used Support Vector Machines (SVM), Naive Bayes (NB), Decision Tree (DT), and K nearest neighbor (KNN) models. For comparison, the authors used the McOne, Relief F, and mRMR FS methods. In their analysis, they used metrics such as accuracy, sensitivity, and specificity. More than 7 to 6 data sets produced satisfactory sensitivity and specificity scores of around 78%. In terms of accuracy, the proposed method only works well on the All, Gastric, Leuk, and Lymp data sets. Reza Rabiei et al.^11^ suggested a method for using ML to classify breast cancer using demographic, laboratory, and mammographic data gathered from the Motamed Cancer Institute in Tehran. The authors in this study employed Multi-Layer Perceptron (MLP), Random Forest (RF), Gradient Boosting Tree (GBT), and genetic algorithm (GA) methodologies. RF has shown better performance than other ML techniques, with accuracy, sensitivity, and specificity values of 80%, 95%, and 80%. Due to a lack of access to genetic data, they have not applied any FS technique for ASSS or modeling based on records from a single database. Yu Shaode et al.^12^ made a mixed system that used four sets of data (BCDR-F03, WDBC, GSE10810, and GSE15852) to test how stable the feature ranking behavior was and how well they could diagnose cancer. They evaluated distinct feature ranking algorithms on four BC datasets and identified three algorithms with excellent stability. The generalized Fisher score (GFS) led to state-of-the-art performance. They evaluated the accuracy and performance of popular classifiers such as SVM, KNN, LDA, NB, and ANN using metrics such as accuracy, sensitivity, specificity, F-measure, and Matthew Correlation Coefficient (MCC). SVM and NB models only performed well on the majority of the four data sets. However, the proposed model only accepts quantitative feature input. Shaode Yu et al.^13^ introduced a study to analyze and evaluate the stability of ranking algorithms using BC data. The researchers computed the stability of the data using sampling, confidence intervals, and hypothesis tests. The levels of stability determined based on the conditions 0.75 represent more stability, while $\ge$40 and $\le$0.75 indicate an intermediately stable nature. The authors experimented on four datasets: BCDR-F03, WDBC, GSE10810, and GSE15852. They identified the GFS, PWFP, and LNEC methods as the most consistent performers across all four datasets. Rather than a comprehensive evaluation, the authors explored the stability of ranking methods using one estimator, which may not be a good idea and seems not to be convincing.

Chun-jiang Tian et al.^14^ developed a unified framework for evaluating the effectiveness of ten FS algorithms using mammographic BC data. The methods used are CFS, ECFS, ILFS, LAPLACIAN, LASSO, LLCFS, RELIEFF, ROC, UFSOL, and Wilcoxon, with a digital database of 104 benign and 980 malignant lesions. They utilized the RF classification method in their study and checked performance using the metrics AUC, ACC, SEN, and SPE. Their study revealed that the correlation, infinite latent FS, exhibited superior performance. They only applied a single classifier, considered a smaller number of features, and primarily used filter-based methods for analysis. Eskandar Taghizadeh et al.^15^ devised a strategy known as a hybrid ML system that incorporates four FS (ANOVA, MI, LR, and Extras Tree classifier) methods. The authors took TCGA data, which contains 762 breast cancer patients and 138 solid tissues, and created three sets of ML algorithms using the 13 classification algorithms they included in their study. The LR method, as a FS and MLP classifier combined, has achieved an accuracy of 86% and an AUC of 94%. Sara Ibrahim et al.^16^ experimented with a methodology for breast cancer diagnosis using correlation analysis and PCA. They implemented the proposed approach using a WDBC set and seven ML classifiers. The proposed approach obtained better accuracy, precision, and recall values. Nahum Cueto Lopez et al.^17^ formulated a system to evaluate different FS techniques using BC data from the MCC Spain study. They used Relief, recursive feature elimination (RFE), and Pearson FS with LR, SVM, MLP, and KNN classification algorithms. In terms of ROC, the features selected with the SVM combination of RFE and the RF model have shown better performance.

To study and analyze over-metastasis BC data, Mahendran Botlagunta et al.^18^ implemented a methodology for classification systems to diagnose cancer metastases. The experimentation analysis used the Welch t-test for data set significance and ML models (LR, SVM, KNN, RF, DT, GB, and XGB) for classification. Out of all models, the DT classifier showed better accuracy (83%), AUC (87%), and scores. Chour Singh Rajpoot et al.^19^ focused on comparing different ML classifiers using BC data from the UCI repository. They have used correlation-based FS for choosing features and applied distinct algorithms (RF, DT, SVM, ANN, and Hybrid Hopfield Neural Network (HHNN)) for BC classification. Researchers have analyzed the proposed method using accuracy, F1-score, sensitivity, specificity, and precision metrics. The HHNN models outperform with scores of approximately 98%. Moloud Abdar et al.^20^ developed an ensemble model for enhancing the BC diagnosis using Wisconsin Breast Cancer Data (WBCD). They developed two-layer nested ensemble models with voting and stacking techniques. They solely considered the Bayes Net and Naive Bayes methods for analysis, taking into account factors such as accuracy, precision, recall, the F1 measure, and ROC metrics to determine the performance of the model. Both methods have performed with an accuracy of 98%. Ganjar Alfian et al.^21^ proposed a web-based BC prediction study using ML classification algorithms. They have used a combination of SVM and the extra-tree classifier-based FS methodology for performing BC analysis. The authors applied XGBoost, AdaBoost, MLP, SVM, etc. Out of all the methods, the extra-tree classifier with SVM has achieved an accuracy of 80%. Rasool A et al.^22^ introduced a study to enhance the performance of ML models by using the correlation RFE mechanism. For their experimentation, the authors used KNN, SVM, LR, and ensemble classifiers from CWDBC and Breast Cancer Coimbra Dataset (BCCD) sources. The LR with RFE achieved 98% accuracy and a 97% F1-score, while the EC with voting classifier achieved 97.6% accuracy among all classifiers. Muhammet Fatih A.^23^ implemented a comparative study with data visualization and ML concepts for BC diagnosis.The author performed FS using RF search and applied distinct ML strategies, such as LR, KNN, DT, RF, SVM, and NB methods. Out of all these methods, the LR classifier was able to achieve an approximate 98% accuracy. Arslan Khalid et al.^24^ developed a study for BC detection and prevention using WBCD data. The authors employed the following ML techniques: SVM, KNN, RF, SVC, DT, and LR for classification. They processed the data using the standard scaler module of the method, as well as univariate and RFE for FS analysis. The classification models obtained accuracy levels of 96.49%, 93.85%, 92.98%, 92.10%, and 87.71% for RF, DT, LR, KNN, and SVC, respectively.

Despite the significant emphasis on ranking-based FS for BC analysis using single FS ranking techniques in previous works, there has been little priority given to FS-based combined ranking for breast cancer analysis. Relying on a single FS strategy for feature ranking is not a good idea, as it may lead to several subsequent consequences, such as the selection of lower-quality features, which often generate poor results and influence the analyst’s decision-making. The aforementioned works reveal that no author has successfully ranked feature schema using multiple FS methods using certain criteria. Therefore, we propose an integrated-based ranking feature selection methodology, which combines estimated individual FS methods with a computed coefficient value-based ranking. The following sections discuss the implementation process of the proposed concept, the algorithm’s time complexity, the overall structure, and its outcomes.

## Implementation

**Fig. 2:**
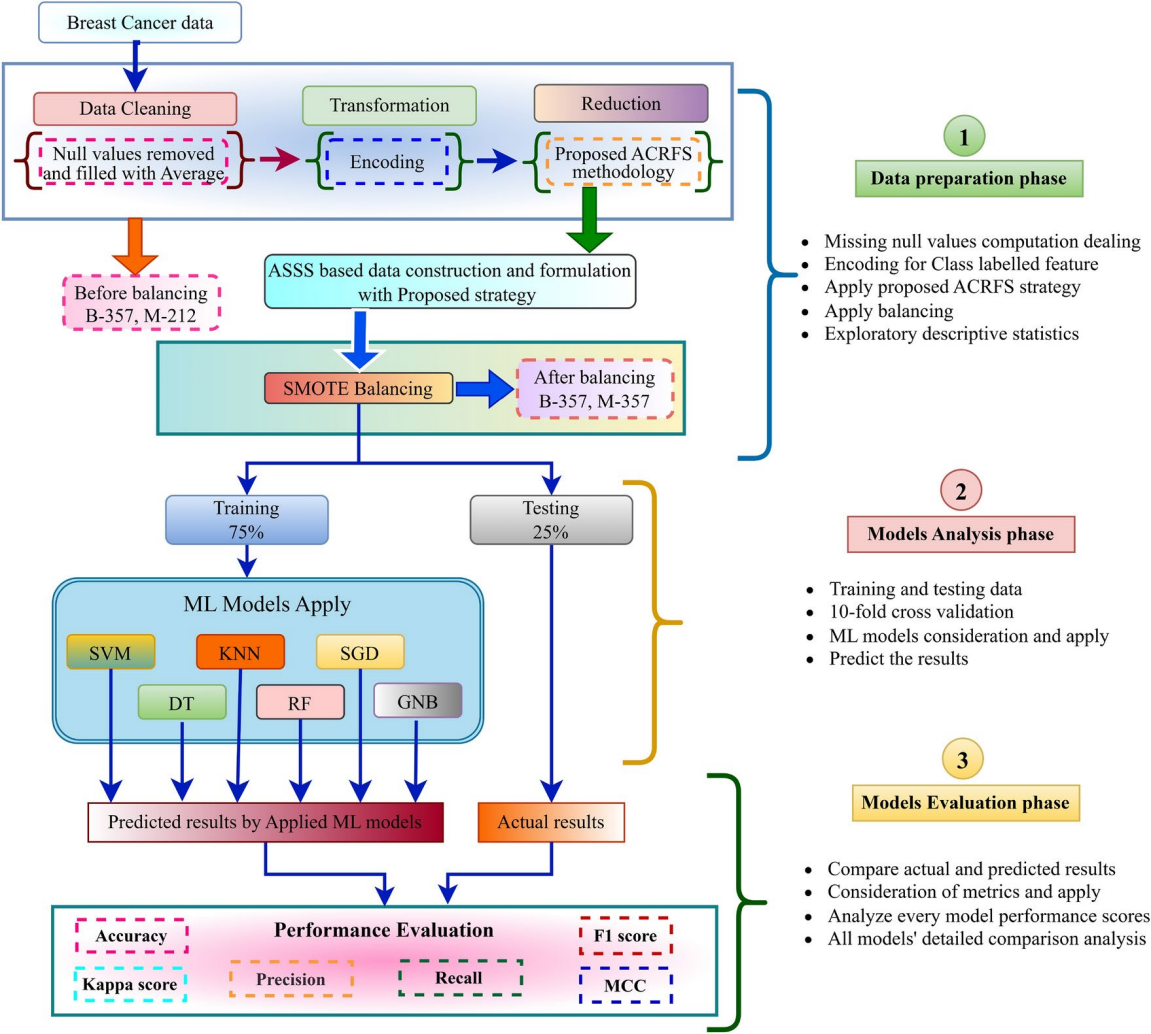
The experimental implementation of representation of complete architecture.

To delve deeper into key component analysis, the experimental system architecture has been divided into three phases to research and analyze breast cancer data, as illustrated in the Fig. 2. This section demonstrates the architecture for forecasting the critical elements of BC analysis using standardized procedures. There are six main parts to the proposed system architecture. These include the repository data set from the University of California Irvine (UCI), cleaning and reduction parts, balancing parts, standardized modules, model analysis and evaluation on each set that is obtained separately, and ranking-based FS and ML method classification results.

### Architecture

**Fig. 3:**
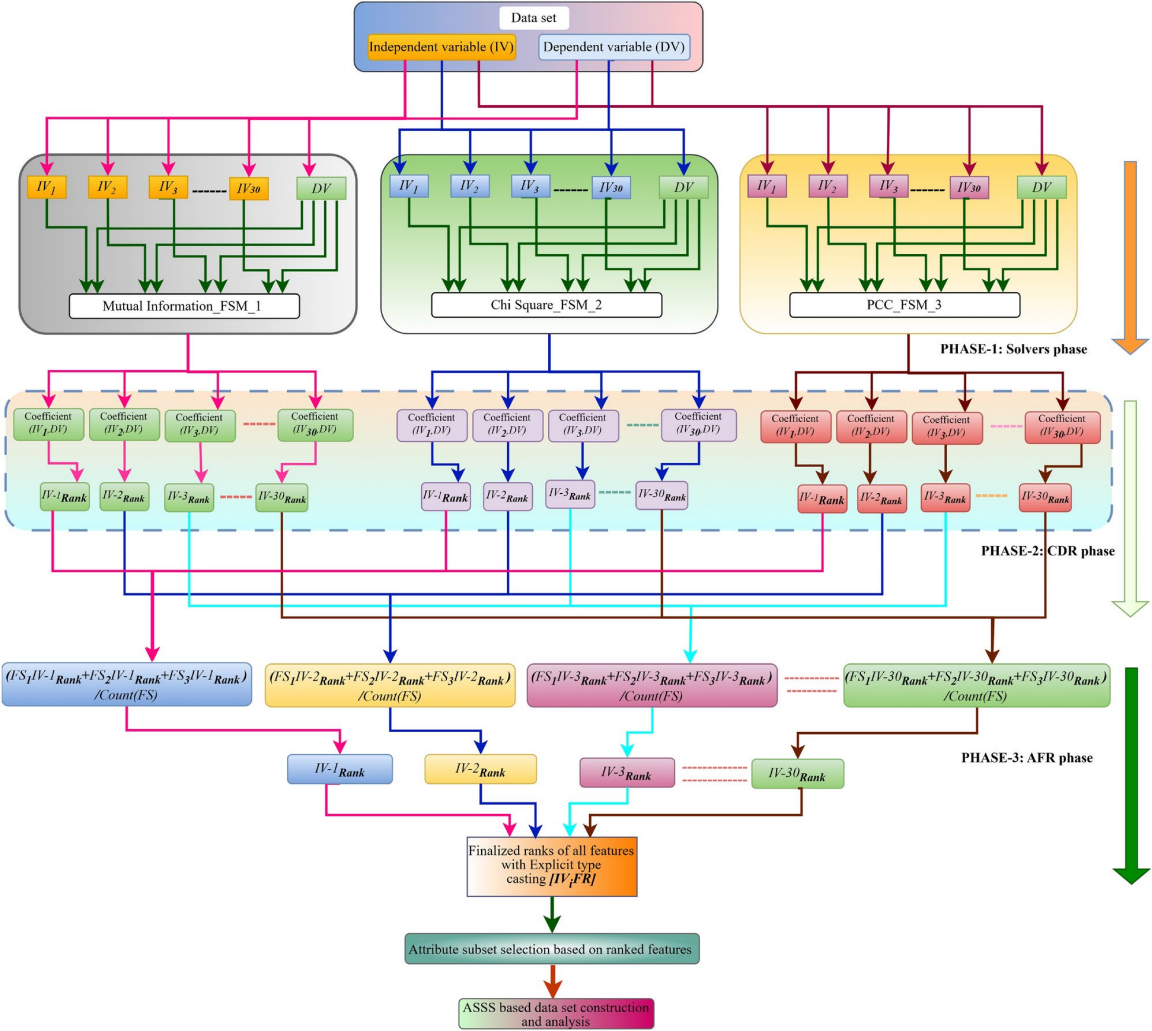
Representation of Proposed Aggregated Coefficient Ranking based Feature Selection strategy.

After transferring the data into an experimental setting, the exploratory analysis characterized using the distribution and dispersion of the data.characteristics are such as central tendency, minimum, maximum, count, mean, interquartile range, and standard deviation. The work has been carried out in three distinct phases: data preparation^25^, model analysis, and model evaluation. The right-side workspace of Fig. 2 provides a clear depiction of the functions of each phase, facilitating simple comprehension. The first stage, known as the data preparation phase, encompasses a sequence of tasks including cleansing, transformation, and reduction. In the vacant locations replaced the null position cell values with average values. Then, transformed the labeled variable, which encompasses both benign and malignant classes, into binary values of 0 and 1. In the reduction step, employed the proposed ACRFS methodology, as depicted in Fig. 3, to identify the most optimal subset. After picking the attribute subset, we created a separate data frame and then conducted SMOTE balancing^8^. SMOTE is a method to address class imbalance in machine learning by creating synthetic samples for the minority class, rather than duplicating existing ones. This improves data balance and helps classifiers learn more effectively. The balanced data was split into 75% training data and 25% testing data in the second phase. The following ML models SVM^36^, KNN^37^, DT^38^, GNB^39^, RF^40^, and SGD^41^ are employed to train data. Previous studies have already delved into a wealth of information regarding the working behavior and principles of ML models. So, we have omitted rather than detailed explanation.

During the third step, a comparison study was conducted between the projected outcomes generated by the models and the actual results. Then assessed each model’s performance by measuring measures such as accuracy, precision, recall, F1-score, Kappa score, and MCC. Subsequently, conducted a thorough comparison and analysis of the computing performance outcomes for all the models in order to ascertain their efficacy.

The reason for considering the methods has been discussed below along with brief explanation of the individual impact of each feature selection method:

**Chi-Square:** This method is particularly useful for categorical data. It evaluates the independence between each feature and the target variable by calculating the Chi-Square statistic. Features with higher Chi-Square values are considered more important because they have a stronger relationship with the target variable.

**Mutual Information:** This method measures the mutual dependence between two variables. It quantifies how much information the presence/absence of a feature contributes to making the correct prediction of the target variable. MI is effective for both continuous and categorical data and can capture non-linear relationships.

**PCC:** This method assesses the linear relationship between two continuous variables. The range spans from -1 to 1, with 1 signifying an ideal positive linear correlation, -1 denoting a perfect negative linear correlation, and 0 indicating the absence of any linear correlation. It helps in identifying features that have a linear correlation with the target variable.

Together, these methods provide a comprehensive approach to FS by capturing different types of relationships and dependencies in the data, which can enhance the performance of machine learning models. For a clear understanding of PCC, MI, and CS methods, the holistic information provided below is discussed in Table 2.

The proposed study was conducted using Google Colab, a cloud-based Jupyter notebook platform optimized for GPU acceleration^26^. Python, renowned for its versatility, was employed to implement the solution, utilizing various modules including Scikit-Learn for ML techniques, Pandas for data analysis, Matplotlib for data visualization, and NumPy for numerical computing^27,28^. These modules provide a flexible and engaging framework for data manipulation and presentation

**Table 2:** The behavioral properties of problem solvers used for coefficient determination and ranking.

Method	Good at dealing with	Coefficient range	Domain category	Data deal with	Prerequisite inputs
PCC	Linear data	-1 to +1	Probability theory	Numeric	Independent variable, Dependent variable
MI	Non-linear data	0 to 1	Statistical measure	Numeric	Independent variable, Dependent variable
Chi-square	Better than PCC and MI	0 to 1	Non-parametric test	Categorical	Independent variable, Dependent variable

### Proposed methodology

As depicted in Fig. 3 the proposed methodological work consists of three phases, namely the problem solver phase, the coefficient determination ranking (CDR) phase, and the aggregated feature ranking (AFR) phase, respectively. During the solver phase, all independent variables are initially categorized as X and the dependent variable as Y. The value_counts() function checks the balance both before and after data assessment to ensure satisfactory balance. The SMOTE technique from the imblearn module is used to achieve a more balanced data distribution. It effectively addresses data imbalances by generating synthetic samples, reinforcing the minority class, and enhancing decision boundaries. The concept considered three algorithms to determine the coefficients for all independent variables with respect to the dependent variable over balanced data. Our approach involved utilizing techniques such as mutual information^29^, chi square^30,31^, and correlation coefficients^32^, respectively. To ensure that the chosen methods are good enough to handle the data in a robust manner for ranking-based FS, We have applied the tri-characteristic behavioral criteria to successfully complete the task. The coefficient solver traits are that MI is great at dealing with nonlinear data, PCC is great at dealing with linear data, and chi square is the best at both finding linear relationships and evaluating nonlinear data.

Subsequently after calculating the coefficients in phase 2, using the rank() and dense approaches to determine the personalized ranks. The rank method applied to assign rankings to the values in each column, and the dense methodology ensures continuous rank assignment without any gaps^33^. After calculating individualized rankings, each independent variable (IV) comprises multiple ranks, which corresponds to the number of problem solvers utilized to compute the coefficients. In Phase 3, we combined all rankings that were based on individual assessments and calculated using different methodologies. We then ranked them according to their respective IV attributes to establish the final rank. This process is known as the aggregated rank. Despite calculating the agg_rank, the rankings remain unordered. We applied the rank() technique to the previously obtained IV wise aggregated values, resulting in the final ranks for each feature. Using the selected features, a new subset of data was generated, ML methods were applied to it, and the model’s efficacy was subsequently evaluated. In contrast to previous techniques, we retained all characteristics, even after picking 72.50% of them. The remaining 27.50% of features have been analyzed and created a hierarchical priority structure for feature categorization, paving the way for further in-depth analysis.

The calculation of mutual information can be determined using the below Eq. 1 shown below.

The mutual information $I(X; Y)$ is defined as:

1 $$\begin{aligned} I(IV; DV) = \sum _{iv \in IV} \sum _{dv \in DV} p(iv, dv) \log \left( \frac{p(iv, dv)}{p(iv) p(dv)} \right) \end{aligned}$$

Where $p(iv, dv)$ represents the joint probability distribution of $IV$ and $DV$ and $p(iv)$ and $p(dv)$ are the marginal probability distributions.

This formulation quantifies the amount of information obtained about one random variable through another, capturing the dependency between them.

The symbol n represents the number of different class values or instances. The chi square coefficients for features can be estimated using mentioned Eq. 2.

2 $$\begin{aligned} {\chi }^2=\sum _{i=1}^{n} \frac{(OV_i - EV_i)^2}{EV_i} \end{aligned}$$

The terms OV and EV represents observed, expected values. The PCC coefficients using below Eq. 3.

3 $$\begin{aligned} PCC = \frac{ \sum _{i=1}^{n} (x_i - \overline{x})(y_i - \overline{y})}{\sqrt{\sum _{i=1}^{n} (x_i - \overline{x})^2 \sum _{i=1}^{n}(y_i - \overline{y})^2}} \end{aligned}$$

After determining the ranks individually finalized rank can be determined using rank method and Eq. 4.

4 $$\begin{aligned} IV[i]\_Agg\_rank= \frac{{\textit{df1\_rank}[i] + \textit{df2\_rank}[i] + \textit{df3\_rank}[i]}}{{\textit{count}(\textit{solvers})}} \end{aligned}$$

### Dataset

**Fig. 4:**
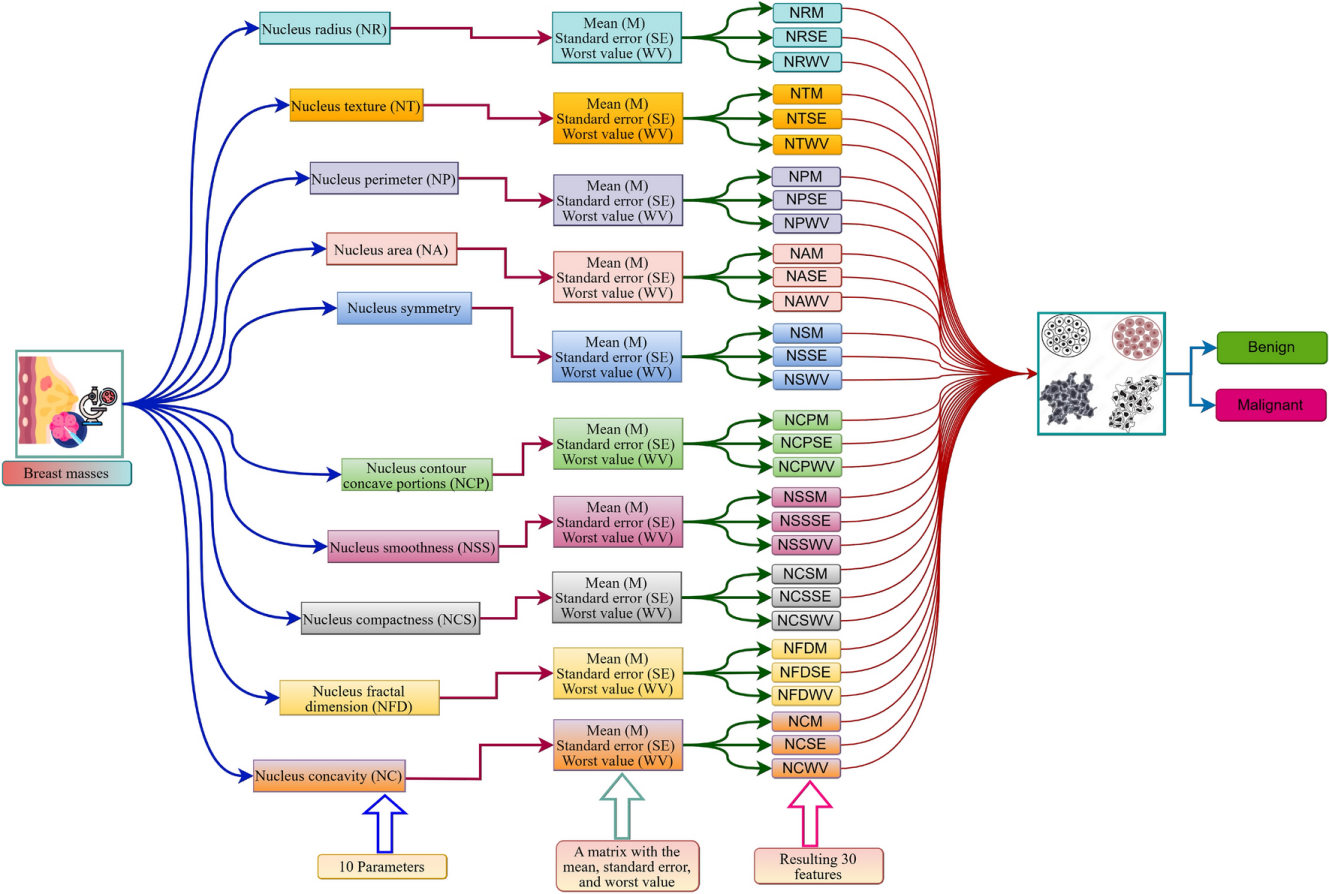
Representation of constructed features from images of FNB based Nuclei Cell characteristics.

The dataset comprises 569 biopsy samples of breast tumors, classified as either malignant (cancerous) or benign (non-cancerous)^34,35^. Digital scans of fine-needle aspirate biopsy slides reveal the characteristics of each sample. The traits correspond to the attributes of the cell nuclei, including their dimensions, morphology, and regularity. They constructed a total of 30 features by determining the mean, standard error, and worst value for each of the 10 nuclear features. Fig. 4 illustrates the data set construction technique for simple comprehension.

### Algorithms design and analysis

The algorithms are specifically designed for study and their analysis. As shown in Table 3 below represents nomenclature and related terms that have been used in the algorithms design Algorithm 1 & Algorithm 2, experimental work, and analysis.

**Table 3:** Notations description used in algorithm design and experimentation.

Nomenclature	
Term	Meaning
AFR	Aggregated finalized rank vector
ASSS	Attribute Subset Selection
CDR	Coefficient Determination Ranking Phase
CS	Chi Square
DV	Dependent Variable
FFFS	Finalized Formulated Feature Set
FSM	Feature Selection Method
IV	Independent Variable
LF	Less influential features
MI	Mutual Information
N	Number of Independent Features
PF	Primary Features
PCC	Pearson Correlation Coefficient
SF	Secondary Features
SP	Solvers Phase
TVR	Threshold Value Rank

**Algorithm 1:**
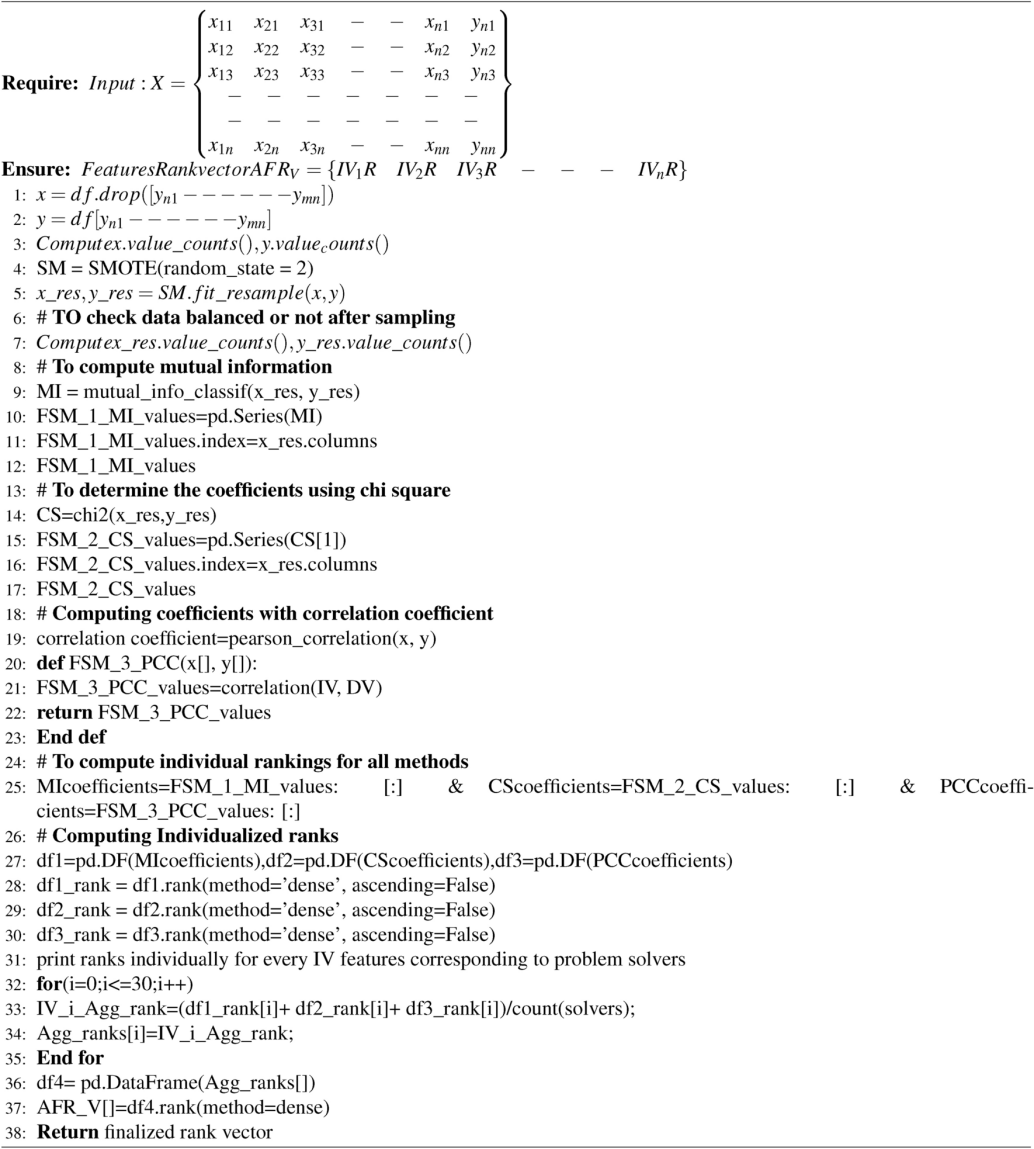
Algorithm for computing the ACRFS based Ranking.

The individual statements would take one unit amount of time for execution. The loop from line 31 to 34 would execute 30 times with respect to input size 30. If there is input Size n the then the worst time complexity of the above algorithm would take $f(n)=\mathcal {O}(n)$.

After constructing the ranking vector, the next step involves creating the finalized data set frame, which is then applied to ML models. The subset formation process uses a threshold rank of 20 as an input, with a ratio of 75%, to select the best features from the ranked vector of features. To achieve the discussed procedure, the algorithm 2 mentioned below will aid us in identifying the appropriate data set.

**Algorithm 2:**
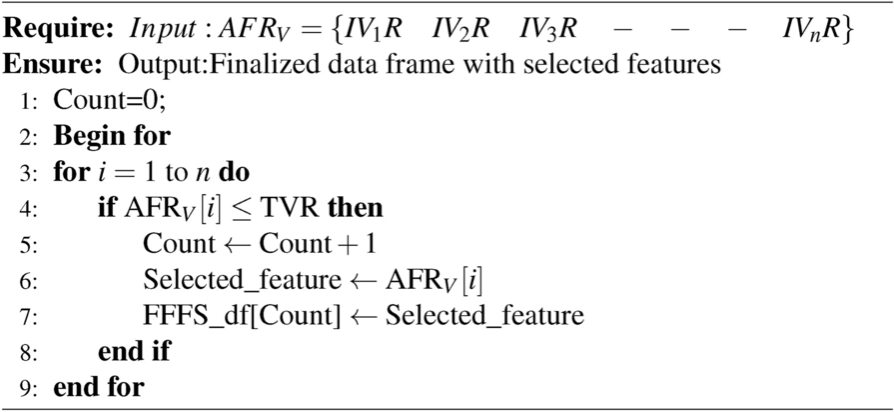
Algorithm for computing the ACRFS based Ranking.

Table 4 provides a step-by-step explanation of the algorithm’s time complexity processing. To analyze the time complexity, examine the feature ranks for the corresponding independent variables, represented in an array with a TVR value of 20, as mentioned below.The computed AFR values for IV, and we consider the TVR value as 20. In the results section end Equation 6 displayed the detailed rules for considering TVR value as 20.

AFR[30] ={1,11,21,2,12,22,3,13,23,4,14,24,5,15,25,6,16,26,7,17,27,8,18,28,9,19,29,10,20,30}.

**Table 4:** Illustrating the computational process for iterative execution behavior of algorithm for determining the time complexity.

For ith loop			If condition and inside statements execution order					Toal No of times executed
Iterations and checking value	Execution status	Count	if(AFR[i] <=TVR)	Execution status	Execution countof if	Execution status	Statement execution count inside if block	=for loop count+ if loop count* (inside if loop statements count)
i=1	P	1	1<=20	P	1	P	1	1+1(1)
i=2	P	2	11<=20	P	2	P	2	2+2(2)
i=3	P	3	21<=20	N	3	Not executes		3+3()
i=4	P	4	2<=20	P	4	P	3	4+4(3)
i=5	P	5	12<=20	P	5	P	4	5+5(4)
i=6	P	6	22<=20	N	6	Not executes		6+6()
i=7	P	7	3<=20	P	7	P	5	7+7(5)
i=8	P	8	13<=20	P	8	P	6	8+8(6)
i=9	P	9	23<=20	N	9	Not executes		9+9()
i=10	P	10	4<=20	P	10	P	7	10+10(7)
i=11	P	11	14<=20	P	11	P	8	11+11(8)
i=12	P	12	24<=20	N	12	Not executes		12+12()
i=13	P	13	5<=20	N	13	P	9	13+13(9)
i=14	P	14	15<=20	P	14	P	10	14+14(10)
i=15	P	15	25<=20	N	15	Not executes		15+15()
i=16	P	16	6<=20	P	16	P	11	16+16(11)
i=17	P	17	16<=20	P	17	P	12	17+17(12)
i=18	P	18	26<=20	P	18	Not executes		18+18()
i=19	P	19	7<=20	P	19	P	13	19+19(13)
i=20	P	20	17<=20	P	20	P	14	20+20(14)
i=21	P	21	27<=20	N	21	Not executes		21+21()
i=22	P	22	8<=20	P	22	P	15	22+22(15)
i=23	P	23	18<=20	P	23	P	16	23+23(16)
i=24	P	24	28<=20	N	24	Not executes		24+24()
i=25	P	25	9<=20	P	25	P	17	25+25(17)
i=26	P	26	19<=20	P	26	P	18	26+26(18)
i=27	P	27	29<=20	N	27	Not executes		27+27()
i=28	P	28	10<=20	P	28	P	19	28+28(19)
i=29	P	29	20<=20	P	29	P	20	29+29(20)
i=30	P	30	30<=20	N	30	Not executes		30+30()
—	—		—			—		—
if input sizeis n then ithloop iterates n times so i=n	n times will execute		n times will execute			Executes (n-TVR+ (n-TVR))		Total execution time =n+n((n-TVR+ (n-TVR)))

Time complexity for algorithm is going to be

$$\begin{aligned} Time complexity:\hspace{5mm} f(n)&= n + n(n - \text {TVR} + (n - \text {TVR})) \\&= n + n(2n - 2\text {TVR}) \\&= n + n(2(n - \text {TVR})) \\&= n + 2n(n - \text {TVR}) \\&= n + 2n^2 - 2n\text {TVR} \end{aligned}$$

$$f(n) = n + 2n^2 - 2\cdot n \cdot 20 \quad \text {(Note: Based on the considered TVR value)}$$

$$f(n) = 2n^2 + n - 40n$$

As per algorithmic analysis after ignoring all the constant values the higher order growth term is ${n^2}$ so The total worst time complexity for an algorithm is

5 $$\begin{aligned} f(n)=\mathcal {O}(n^2). \end{aligned}$$

## Results

This section presents the outcomes of the proposed ACRFS approach, along with comprehensive data analysis and conclusions. The methodologies used for the BC dataset and their results are visually displayed, simplifying the understanding and identification of the range of coefficient values and the derived conclusions. To estimate the critical factors for BC diagnosis, we use a standardized process that employs mutual information, chi-square, and coefficient solvers. Next, we apply dense and rank methods to assign significant rankings to the features, based on the computed coefficients determined by the problem solvers. Subsequently, the selected subset was framed, and the following methods were applied: RF, GNB, DT, KNN, SVM, and SGD. Following the prediction outcomes, we conducted performance and evaluation analyses to showcase the model’s effectiveness.

**Table 5:** The problem solvers obtained coefficients and associated ranks for features.

Features	Mutual_info		Chi square		PCC		Aggregated rank	Finalized Rank
	Coefficient	Rank	Coefficient	Rank	Coefficient	Rank		
NRM	0.389408	9	3.96E-77	24	0.73	7	13	9
NTM	0.142787	17	4.83E-26	21	0.41	19	19	25
NPM	0.410619	6	0.00E+00	26	0.74	5	12	7
NAM	0.399259	8	0.00E+00	26	0.70	8	14	11
NSM	0.08718	23	6.58E-01	6	0.35	21	17	17
NCSM	0.260138	14	1.04E-02	14	0.59	11	13	8
NCM	0.407729	7	4.24E-06	17	0.69	9	11	4
NCPM	0.476798	3	7.56E-04	15	0.77	3	7	1
NSM	0.085045	25	5.39E-01	9	0.33	22	19	23
NFDM	0	30	9.85E-01	2	-0.0	29	20	28
NRSE	0.269881	13	1.12E-10	19	0.56	13	15	13
NTSE	0.008515	29	8.55E-01	5	-0.0	28	21	30
NPSE	0.271722	12	2.39E-66	23	0.55	14	16	16
NASE	0.356889	11	0.00E+00	26	0.54	15	17	18
NSSE	0.014654	28	9.46E-01	3	-0.0	30	20	28
NCSSE	0.103417	22	3.90E-01	11	0.29	24	19	25
NCSE	0.142333	18	2.94E-01	12	0.25	25	18	21
NCPSE	0.157246	16	5.73E-01	8	0.40	20	15	12
NSSE	0.024105	27	9.96E-01	1	-0.0	27	18	21
NFDSE	0.052852	26	9.36E-01	4	0.07	26	19	23
NRW	0.474301	5	1.30E-136	25	0.77	4	11	5
NTW	0.141857	19	1.70E-47	22	0.45	16	19	25
NPW	0.505403	1	0.00E+00	26	0.78	2	10	3
NAW	0.480071	2	0.00E+00	26	0.73	6	11	5
NSW	0.114092	20	4.72E-01	10	0.42	17	16	15
NCSW	0.241209	15	2.32E-06	18	0.59	12	15	13
NCW	0.366975	10	8.20E-11	20	0.65	10	13	9
NCPW	0.47445	4	1.28E-04	16	0.79	1	7	1
NSW	0.112169	21	1.80E-01	13	0.41	18	17	18
NFDW	0.086464	24	6.00E-01	7	0.32	23	18	20

Table 5 represents the calculated coefficient scores for MI, chi square, and coefficient methods and the finalized ranks. The computed coefficient values will assist us in selecting the optimal features for conducting analysis. We have assigned ranks to the features based on the computed coefficients. The effectiveness of the proposed methodology has been verified through the following computational procedures:

- The performance of the applied ML models was evaluated by comparing their performance before and after implementing our methodology, and the results were clearly demonstrated.
- The current works of researchers on FS based on ranking concepts have been compared with their respective results and our own implemented outcomes, which align with our objectives.

**Fig. 5:**
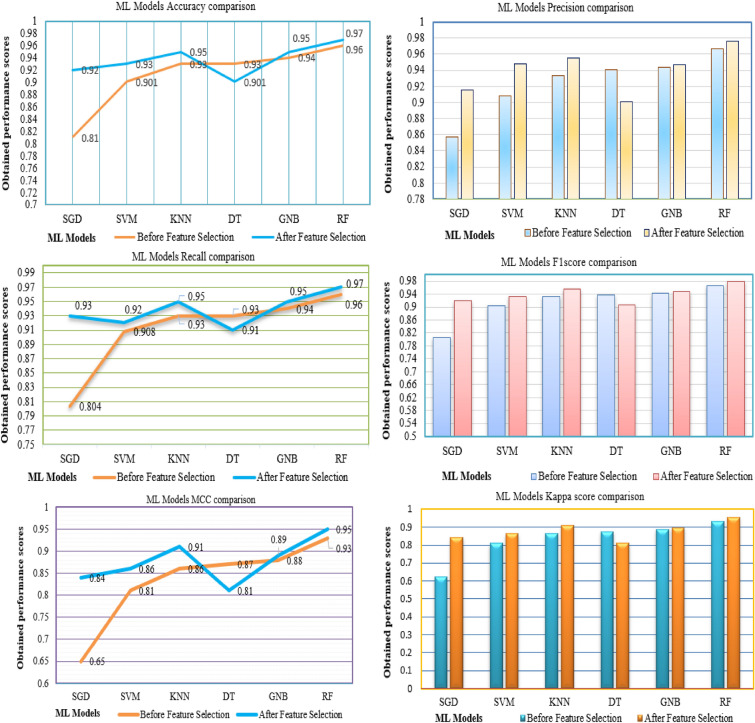
The graphs represents obtained scores by distinct ML models before and after FS.

Table 6 and Fig. 5 show the performance scores achieved by several ML models prior to and after implementing our suggested methodology. We carry out the performance evaluation and analysis by computing metrics such as accuracy, precision, recall, F1 score, kappa score, and MCC. Furthermore, the subfigures illustrate the improved scores achieved by implementing our methods. As shown in Fig. 5 it can be observed that the performance of evaluated models has improved after FS, with the exception of DT model and allows us to observe, draw a few key conclusions.

- The proposed methodology has yielded favourable results in terms of all classification metrics when using SVM, KNN, and RF. Overall, SGD has demonstrated superior performance, with a 19% increase in potential and significant improvements in metrics such as MCC and kappa score. Additionally, there have been 11% increases in accuracy, recall, and F1-score, as well as a 6% increase in precision levels.
- The performance of GNB has been carefully examined and significantly improved its potential levels, resulting in an improvement of more than 2% across all metric scores.
- However, it is noteworthy that the DT has exhibited anomalous behaviour towards our suggested approach, resulting in a decline of 2% in accuracy, recall, F1 score, and kappa score. Additionally, there has been a fall of 4% and 6% in precision and MCC respectively.
- The proposed concept was well in line with the substantial improvement in performance achieved by SGD. Furthermore, the precision levels exhibited by RF showed exceptional performance, resulting in a staggering accuracy score of 97%. The models SVM, KNN, and GNB have shown enhanced performance in attaining a balanced and elevated level of outcomes. The DT strategy has demonstrated inadequate resilience when confronted with a decrease in performance levels resulting from negative conduct.

**Table 6:** The comparative results with and without ACRFS methodology.

Model	Before Feature Selection						After Feature Selection					
	Accuracy	Precision	Recall	F1 score	Kappa score	MCC	Accuracy	Precision	Recall	F1 score	Kappa score	MCC
RF	0.9664	0.9663	0.9663	0.9663	0.9327	0.9327	0.979	0.9763	0.9795	0.9779	0.9558	0.9559
GNB	0.9441	0.9437	0.9453	0.944	0.8881	0.889	0.951	0.947	0.9515	0.9484	0.8969	0.897
DT	0.9385	0.941	0.9365	0.938	0.8762	0.8775	0.909	0.9008	0.9159	0.9059	0.8124	0.8166
KNN	0.9329	0.9331	0.9347	0.9329	0.8659	0.8679	0.958	0.9556	0.9556	0.9556	0.9113	0.9113
SVM	0.905	0.9084	0.9084	0.905	0.8107	0.8169	0.937	0.9474	0.9215	0.9317	0.8637	0.8686
SGD	0.8156	0.8568	0.8049	0.8059	0.6223	0.6597	0.923	0.9155	0.934	0.9208	0.8423	0.8494

### Comparison results

Table 7 represents considered works for comparing the proposed work to prove its effectiveness levels and also shows the accuracy results obtained by various approaches with ML models applied by different authors. Sara Ibrahim et al.^16^ implemented distinct ML methods using hard and soft voting approaches, and they were able to achieve approximately 97% accuracy with the LR model. Ganjar Alfian et al.^21^ experimented with their work using various ML classification techniques such as XGBoost, AdaBoost, MLP, LR, KNN, DT, NB, RF, and SVM with Extra-Trees. Out of these methods, SVM with Extra-Trees has better accuracy (80%), precision (82.71%), sensitivity, and specificity (78.57%). Rasool A et al.^22^ implemented a voting classifier model, and they were able to achieve accuracy of 97.67% and 96% of the F1 score. Muhammet Fatih A^23^ implemented SVM, KNN, RF, DT, and LR models with all features, and he was able to achieve 98% accuracy with LR, but it requires more storage and time complexity. Arslan Khalid et al.^24^ developed methodology by employing a soft voting classifier (SVC) along with LR, NB, RF, XGB, NB, and DT algorithms, achieved a good accuracy rate of 96.49%.

Table 7 clearly demonstrates that the proposed approach, when compared to other state-of-the-art works, performs less effectively in terms of precision, F1 score, sensitivity, and specificity. The proposed methodology, utilizing ML techniques, achieved the highest F1 score (97.79%), precision (97.63%), and recall (97.75%). The method successfully selected crucial features, enabling the models to train more effectively and with less computational complexity. The proposed method achieved approximate accuracy with two works, but in terms of precision and recall, the F1score has shown significant improvement.

**Table 7:** The comparative results with other relevant works.

Authors	Models applied	Results
Sara Ibrahim et al.^16^	SVM, KNN, DT, NB, RF, XGB, LR	Approximate 97% accuracy, precision, recall by LR
Ganjar Alfian et al.^21^	AdaBoost, MLP, LR, KNN, DT, NB, RF, SVM	Accuracy (80%), precision (82.71%), sensitivity & specificity is (78.57%)
Rasool A et al.^22^	Voting Classifier	Accuracy (97.6%), F1 score (96%)
Muhammet Fatih A^23^	SVM, KNN, RF, DT, LR model with all features	Accuracy (98%)
Arslan Khalid et al.^24^	SVC, KNN, DT, RF, LR, DT	Accuracy (96.49%)
Proposed concept	ACRFS with RF, GNB, DT, KNN, SVM, SGD	Accuracy (98%), F1 score (97.79%), precision (97.63%), recall (97.75%)

We have outlined our hierarchical organization selection categorization criteria below for easy comprehension. We have categorized the dataset features into three hierarchical groups: primary features, secondary features, and less influential features. We have formulated and utilized the following criteria to categorize the features into primary, secondary, and less influential groups. These equations will assist in determining the category to which a feature belongs based on its rank. The function below provides a clear and effective way to categorize features based on their ranks.

Below criteria have been followed to formulate the feature category:

6 $$\begin{aligned} r(iv,dv)_i(n) = {\left\{ \begin{array}{ll} 72.5\% & \text {if } i \le r(iv,dv)< 20 \\ 13.25\% & \text {if } 20 \le r(iv,dv)< 25 \\ 13.25\% & \text {if } 25 \le r(iv,dv) < n \end{array}\right. } \end{aligned}$$

Where i=1,2,3,,,,,,n-2, n-1,n and n indicates the ranks for IV features in the data set.

**Primary Features:** These are the most important features, accounting for 72.5% of the total. Their ranks range from 1 to 19. We refer to the PF set of primary features, which comprise 72.5% of the total features and have ranks ranging from 1 to 19 (inclusive).

**Secondary Features:** These features are moderately important, making up 13.25% of the total features. They rank from 20 to 24. We refer to the set of secondary features (SF), which comprise 13.25% of the total features and have ranks between 20 and 23 (inclusive).

**Less Influential Features:** These are the least important features, also making up 13.25% of the total features. They rank between 25 and 29. Less Influential Features (LIF) make up 13.25% of the total features and rank between 25 and 29 (inclusive).

**r (iv, dv):** This represents the position or importance of a feature within the data set. We use the rank to categorize features into primary, secondary, and less influential groups.

The study has progressed through a series of steps, including data preparation, statistical features analysis, FS methods, and critical factor analysis for BC analysis using ML models. Having completed the experimental process with the main features, our focus shifted to addressing the remaining features not included in the analysis. Our critical factor analysis has identified the following features as primary key factors: The identified factors include NRRM, NPM, NAM, NSM, NCSM, NCM, NCPM, NRSE, NTSE, NPSE, NASE, NCPSE, NRW, NPW, NAW, NSW, NCSW, NCW, NCPW, and NSW. We have also determined the secondary influential features to be NSM, NCSE, NSSE, NFDSE, and NFDW. Furthermore, our analysis has recognized the remaining features of BC, namely NTM, NFDM, NSSE, NCSSE, and NTW, as the characteristics with the least overall impact.

Further, the work can be extended using deep learning. By utilizing multiple imaging modalities, these studies highlight the potential of combining computational methods to enhance the accuracy of medical breast cancer diagnoses. Integrating multiple data repositories, the referenced studies can also assist in carrying out the research effectively^42–44^. Additionally, the work can be extended by considering the innovative optimization techniques Greylag Goose Optimization^45^ and Puma Optimizer^46^ which can significantly enhance feature selection in machine learning tasks.

### Limitations

- The time complexity of the proposed ACRFS approach increases with the number of solvers used. Each additional solver adds more computational work, which means more time is needed to complete the process. While using more solvers can improve the accuracy and robustness of the feature selection, it also requires more computational resources. Therefore, it’s important to balance the number of solvers to ensure that the benefits outweigh the increase in time complexity.
- To accurately determine the number of solvers for coefficient determination strategies, we employed a generic process rather than any specific computational method. This approach ensures a broader and more flexible assessment.
- The data used for our analysis was mostly numerical, with just a few labeled or class-specific variables. Importantly, there wasn’t much categorical data. This focus on numerical data made our analysis more straightforward and reliable. The lack of diverse data types simplified the process, ensuring clear and precise results. This careful selection of data types contributed significantly to the clarity and robustness of our findings.
- When conducting analysis for clinical applications by integrating data from multiple sources, it is crucial to ensure that the model utilizes the integrated data with common characteristics for seamless interpretability. This requires careful consideration and alignment of the data’s attributes to ensure consistency and accuracy. By standardizing the data characteristics, the analysis becomes more reliable, and the results can be interpreted with greater ease, ultimately enhancing the clinical decision-making process. It is imperative to meticulously harmonize the data inputs so that the model can effectively process and provide meaningful insights.

## Conclusion and future work

A comprehensive analysis of all cancer types was performed, taking into account cancer-related data. The study revealed that BC had the highest impact on mortality. The aim is to tackle numerous BC-related concerns by conducting an efficient FS analysis using multiple-condition criteria. This article delves into this work, unveiling a new method for FS ranking to perform effective BC analysis using ML based on coefficient determination analysis for features. The suggested ACRFS method was implemented using dense, aggregated computing techniques with three problem solvers, such as MI, CS, and PCC. The introduced method was applied to establish a new ASSS mechanism over WBCD data. Machine learning models were deployed on the constructed subset of data, and outcomes were examined using various classification metrics. The proposed concept progressed swiftly, with ACRFS demonstrating an impressive exponential performance increase with SGD, and RF outperforming SGD in precision levels, achieving a high 97% accuracy. Results demonstrated appropriate equilibrium performance enhancement levels using SVM, KNN, and GNB. The DT method showed a lack of resilience when performance levels declined due to negative behavior. The results and comparison sections clearly show that the proposed concept model outperforms all listed models, demonstrating the highest performance levels and outperforming other existing methods. Coefficient determination, ranking, and feature selection are interdependent. The study focused on exploring multi-criteria-based feature ranking to obtain a suitable set for analysis. Future advancements can be made by intensifying the focus on coefficient estimation analysis, a method that rigorously analyzes the feature coefficients, potentially improving ranking and feature selection for attribute subset formation. This strategy could effectively enhance all associated characteristics of the analysis process, making it easier to interpret and analyze.

## Data Availability

The dataset we have utilized for the proposed methodology has been taken from the Machine Learning repository [https://archive.ics.uci.edu/dataset/17/breast+cancer+wisconsin+diagnostic], [https://doi.org/10.24432/C5DW2B].
